# Is Birth Method Associated with Sensory Hyperreactivity in Children 3-4 Years in an Upper-Middle-Income Country?

**DOI:** 10.1155/2023/5598392

**Published:** 2023-11-25

**Authors:** Ann Frances Watkyns, Pamela Joy Gretschel, Helen Buchanan

**Affiliations:** ^1^10 Kings Way, Pinelands 7405, South Africa; ^2^Division of Occupational Therapy, Department of Health and Rehabilitation Sciences, University of Cape Town, Cape Town, South Africa

## Abstract

**Background:**

Research has shown that pressure in the vaginal birth process aids the infant's neurophysiological adaption to extrauterine life, including their ability to regulate their responses to diverse sensory stimuli. As pressure is absent in elective caesarean section births, we hypothesised that these children may be at higher risk for developing sensory hyperreactivity (SHR), a sensory modulation difficulty which negatively impacts on the child's engagement in their occupations. This paper reports on a study which investigated associations between birth method and SHR.

**Method:**

In this cross-sectional study, 91 children aged three and four years from various language, cultural, and socioeconomic groups were recruited and categorised based on birth method (caesarean section or vaginal birth). Caregivers of each child completed the Short Sensory Profile-2 and a demographic questionnaire. The prevalence of SHR between the birth method groups was compared.

**Results:**

The prevalence of SHR was greater in the vaginal birth group (29%) than the caesarean section group (9%). Mothers in the vaginal birth group were younger (*p* ≤ 0.001), of lower-income level (*p* = 0.003), and more likely to be single (*p* = 0.037). During data collection with the vaginal birth group, comprehensibility of certain items in the Short Sensory Profile-2 proved challenging for many caregivers.

**Conclusion:**

The statistically significant higher prevalence of SHR in the vaginal birth group supported a null hypothesis. However, the results are questioned due to the challenges related to data collection. Given this, the study supports the need for further investigation of how sociocultural and socioeconomic factors influence the assessment of SHR in the South African context.

## 1. Introduction

Sensory hyperreactivity (SHR) is an overresponsive behavioural reaction to nonharmful, nonthreatening sensory stimulation, which can have a profound effect on the child's emotional status, behaviour, sleep, concentration, and socialising, impacting on their engagement in the occupations of school, play, and self-care [1]. The causes of SHR are not known, although it has been proposed that prematurity and prenatal complications may be factors [2, 3]. Anecdotal evidence from the first author's practice experience, as well as that of colleagues, and a recent research study [4] indicate a marked increase in sensory modulation disorder and particularly SHR in recent years. Children with SHR are frequently treated by occupational therapists, who use the application of deep pressure as an important component of their therapy [5].

The caesarean section (CS) rate has increased both worldwide [6] and in South Africa [7, 8]. Health care in South Africa is divided into two sectors largely determined by financial means. A private health sector caters primarily for those on a private medical aid and a public health sector for those with no medical aid, who tend to be of lower socioeconomic status. In the private health sector of South Africa, the CS rate was almost 70% for those on medical aid [8], compared to an average 24.1% in the public sector [9]. The World Health Organization has determined acceptable CS rates of between 10 and 15% of deliveries, for cases where medical intervention is necessary [10]. Doctors may choose to perform a CS for their convenience, fear of litigation, or medical concerns related to excessive maternal weight gain and increasing maternal age [11]. Possible reasons for the mother include fear of childbirth, a perception that CS is a safer option and CS becoming an increasingly common trend [11, 12]. One feature of an elective CS is that the baby does not experience the pressure associated with a vaginal birth (VB).

Pressure is a component of a VB resulting from uterine contractions and the passage down the birth canal. Research on rats linked the pressure experienced during vaginal birth to effective neurophysiological adaption (respiration and suckling) in extrauterine life [13]. In humans, the pressure causes activation of the sympathoadrenal axis, with release of noradrenaline, vasopressin, and other hormones [14]. These hormones have various effects during the perinatal period on the mother (facilitate maternal behaviours and stimulate milk production) and on the infant (aid lung maturation, infant alert state, and olfactory recognition of the nipple). The possible influence of birth method has been explored in both mothers and infants. Mothers who had a CS were reported to have higher breastfeeding failure due to decreased release of noradrenaline, oxytocin, and other hormones [15]. Mothers who had a VB had heightened maternal neural responses to the sound of their infant's cry [6]. Maternal regulation of the infant occurs chiefly through breastfeeding and skin-to-skin contact in the immediate postpartum period, which is the basis for the development of successful infant self-regulation [16–18]. Increased breastfeeding failure and reduced maternal responsiveness to the infant's cry in CS mothers may reduce the mother's ability to provide this maternal regulation, negatively impacting the development of infant self-regulation. Slower visual attention in infants born by CS has been reported [19]. In later life, children born by CS were reported to have a statistically significant increased risk of developing asthma [12, 20], obesity [12], autism spectrum disorder, and attention deficit hyperactivity disorder [21]. The link between infant self-regulation and SHR seems evident; however, no literature could be found to confirm this link.

Associations between SHR and socioeconomic status were explored in a large study investigating the prevalence of, and risk factors for, SHR in children of seven to eleven years [22]. The study found birth weight, gestational age, minority ethnicity, living with a single and/or unemployed parent, and low socioeconomic status to be significant risk factors associated with the development of SHR [23]. These results were however not supported by Román-Oyola and Reynolds who examined sensory modulation, not only the SHR component, in Puerto Rican preschool children of varied economic status [24]. Although they found a higher prevalence of sensory modulation disorders than in the United States, they found no significant difference in prevalence of sensory modulation based on socioeconomic status [24]. However, there were statistically significant differences in two aspects of sensory modulation, namely, movement sensitivity (vestibular SHR) and hyporeactivity. The sample size was however small (*n* = 141), limiting the generalisability of the results [24].

Our literature search highlighted that the aetiology of SHR remained unknown. Anecdotal evidence supported a single study reporting an increase in SHR. Evidence on the relationship between socioeconomic status and SHR was inconclusive. Speculations about the relationship between the increased rate of CS and the increase in SHR led us to explore this relationship further. This study tested whether method of birth was associated with SHR. Based on evidence from the animal and human studies reported above, it was hypothesised that there would be a higher prevalence of SHR in children born by elective CS compared to those born by VB.

## 2. Method

### 2.1. Study Design

A cross-sectional study design was used. Ethical approval was obtained from the University of Cape Town Faculty of Health Sciences Human Research Ethics Committee.

### 2.2. Sampling

The study took place in Cape Town, a city of South Africa. The sample consisted of children aged three and four years old attending early childhood development (ECD) centres and their caregivers. The required sample size, calculated using the Cochrane formula [25], with a confidence level of 85% and a margin of error of 5%, was 84 (42 in each birth group). The sampling frame was a City of Cape Town list of all registered and nonregistered ECDs in three neighbouring areas of Cape Town, totalling 86 ECDs. These areas represented three household income levels based on the 2011 census figures [26] (hereafter referred to as “high,” “middle,” and “low income” groups). This point of recruitment provided the best opportunity to access a broad group of children potentially from different socioeconomic backgrounds, cultures, and ethnicities. Child inclusion criteria were a singleton pregnancy and, in the case of VBs, birth by cephalic presentation. Exclusion criteria were serious confounding life events (such as death of a parent), emergency CS, presence of cerebral palsy, orthopaedic conditions, Downs syndrome, an IQ below 85, global developmental delays, and previous occupational therapy. The caregiver needed to have been the child's caregiver for the past 12 months.

The sampling procedure occurred at two levels. The first level involved selection of ECDs. The first author allocated each ECD in the sampling frame an ascending number from 1 to 86. To avoid selection bias, the second author, who did not have access to the sampling frame, selected 12 computer-generated random numbers between 1 and 86. These were shared with the first author who linked these numbers to the ECD on the numbered list. The management of the selected ECDs was approached, and written consent was provided to conduct the research. The second level of sampling was of child-caregiver dyads at each ECD. Teachers of children of the correct ages were provided with information letters and reply slips to give to parents. These letters requested participation in the study. The first author collected reply slips a week later and handed them to the research assistant who made telephonic contact with each caregiver to check that both caregiver and child met the selection criteria. Following telephonic consent from the caregivers, the research assistant requested information about the child's birth method. As parental consent was received, the research assistant allocated an ascending number starting from 1 to each child-caregiver dyad. In addition, a number was allocated based on the child's birth method, with 0 representing the VB group and 1 representing the CS group. The child's study number was used to ensure blinding of the first author to the child's identity and birth method.

The recruitment process was completed at all the selected ECDs without obtaining sufficient numbers of participants and with fewer participants in the high-income level group compared to the middle and low groups. The sampling process was repeated in ECDs from the high-income level group only, to increase the number of participants in this group and ensure adequate representation of all income levels in each group [27].

### 2.3. Data Collection Tools

*The Short Sensory Profile 2^nd^ edition* (SSP-2) [5], a 34-item parent questionnaire designed to measure behaviours associated with abnormal responses to sensory stimuli in children aged 3.0–14.11 years, was chosen to determine the prevalence of SHR in the sample. The SSP-2 is theoretically informed by Dunn's sensory processing framework which has four quadrants describing the child's neurological threshold to sensory input and their method of self-regulation [5]. The four quadrants are seeking (7 items), avoiding (9 items), sensitivity (10 items), and registration (8 items). Cronbach's alpha indicates good internal consistency for each quadrant (seeking: *α* = .69; avoiding: *α* = .83; sensitivity: *α* = .75; and registration: *α* = .75) [5]. The behaviour the child displays for each item is scored on a Likert scale ranging from 1 (almost never = 10% or less) to 5 (almost always = 90% or more). Raw score totals can be calculated for each quadrant. A bell curve distribution based on a normative sample of children without disabilities (*n* = 697) allows for raw score totals for each quadrant to be classified as “much less than others” (lower 2%), “less than others” (between 1 SD and 2 SD below the mean, accounting for 14% of the normative sample), “just like the majority of others” (±1 SD from the mean and accounting for 68% of the normative sample), “more than others” (between 1 SD and 2 SD above the mean), and “much more than others” (upper 2%). This instrument is designed for diagnostic use with children [5], is quick to complete [5], has been used in several studies investigating SHR [28], and is widely used by occupational therapists. The SSP-2 has high interrater and test-retest reliability, moderate to high construct validity, and high content and discriminant validity [5]. The SSP-2 was standardised on American children [5]; therefore, various measures were taken to evaluate its appropriateness and accuracy in the South African context [29]. Six community members were selected, two from each of the three income level areas represented in the study, to reflect the diversity in languages, races, cultures, socioeconomic level, and education across these levels. They read and completed the questionnaire and identified challenges they experienced in its use. Further details are described elsewhere [29, 30].

To explore the impact of other possible casual factors of SHR, *a demographic questionnaire,* informed by all available evidence describing causal factors of SHR, was developed. These included maternal illnesses and stress and birth-related complications [3].

### 2.4. Data Collection

The research assistant emailed the contact details of the recruited participants to the first author without divulging birth method. The first author then scheduled appointments to meet each caregiver to sign the informed consent form after which caregivers completed the SSP-2. The first author was unaware of the birth method and thus blinded at the stage of assessing SHR. Caregivers then completed the demographic questionnaire. In addition, the first author made and recorded informal observations regarding sensory features of the environments, particularly related to auditory, visual, tactile, and olfactory input, and characteristics of children's schedules and routines during data collection. These observations were not systematically coded or included in analyses in any way.

Once data collection commenced, the first author noted that the caregivers' ease of completing the SSP-2 questionnaire varied for each income level [29]. Caregivers in the high-income group understood and completed the questionnaire easily, which appeared to be due to English being their home language and the majority having a high educational level. Caregivers in the middle-income group needed some assistance in the form of requests for clarification on some statements and the meaning of the five scaled divisions in the Likert scale. For many of these caregivers, English was their second language, but they understood and spoke English well. For many of the caregivers in the low-income group, English was their second or third language and comprehension of written and spoken English was frequently more challenging than for the caregivers from the middle-income group. These caregivers experienced similar types of difficulties to the middle-income caregivers, but they occurred more frequently and in some instances could not be resolved. The first author made adaptations while collecting this data in an attempt to increase the accuracy of the responses. Adaptations took the form of additional or alternative wording to aid understanding of complex language and the use of the Likert scale. These challenges did however raise concerns regarding the validity of the data obtained, specifically face and content validity. As a result, data collection was halted after four low-income group caregivers had completed the questionnaire. These challenges were discussed with the research team and further adaptations were made, taking the form of visual aids to enhance understanding of the particularly problematic statements and the Likert scale [29, 30]. As these additional adaptations were similar to those made by the first author during data collection with the four caregivers from the low-income group, the decision was made to include this data in the analysis. Data collection then resumed with the adaptations being applied in a standardised manner when required by caregivers. The researcher found these adaptations to be effective in improving understanding of the questionnaire and increasing accuracy of caregiver responses.

### 2.5. Data Management

The completed, numbered questionnaires and demographic information sheets for each caregiver were collated and placed in a locked box file for transportation to the first author's home where they were stored in a locked filing cabinet for the prescribed period of five years. The first author scored the SSP-2 on the original scoring sheets and calculated raw score totals and standard deviations (SDs) for the six subsections and the four sensory modulation categories. Scores of both a SD of +1 (“more than others”) and +2 (“much more than others”) indicated atypical performance, and zero indicated typical performance [5]. The SD scores for the sensitivity quadrant were used to identify children with SHR as test items related to this quadrant reflected primary behaviours identified by the test developers as being closely aligned to SHR [5]. The other quadrants (avoiding, seeking, and registration) could be attributed to SHR as secondary behaviours, but only indirectly, and could also be linked to other factors. On completion of the scoring for each assessment, the first author entered the individual item scores, total category scores, and category SDs onto a password-protected Microsoft Excel spreadsheet which could be accessed by the first author and research assistant. Checks were performed and corrections made where necessary during all stages of data collection and analysis [27].

### 2.6. Data Analysis

Data were exported from Microsoft Excel into Statistica (StatSoft 2018) for analysis. As data for SHR raw scores were not normally distributed (*W* = 0.951, *p* = 0.002), nonparametric tests were used throughout. Medians and ranges were determined for numerical variables and frequencies and percentages for categorical variables. Associations between demographic variables and birth method group, prevalence of SHR by birth method group, and demographic variables and SHR were determined with Fisher's exact test (two-tailed), chi-squared test of association, or the Mann–Whitney *U* test as appropriate. The confidence interval was set at 85% and level of significance at *p* < 0.05 throughout. As an unexpected statistically significant relationship between socioeconomic status and SHR emerged during the data analysis, we explored the links between socioeconomic status, birth method, and SHR further post-hoc.

## 3. Results

### 3.1. Participant Flow

**Figure 1:**
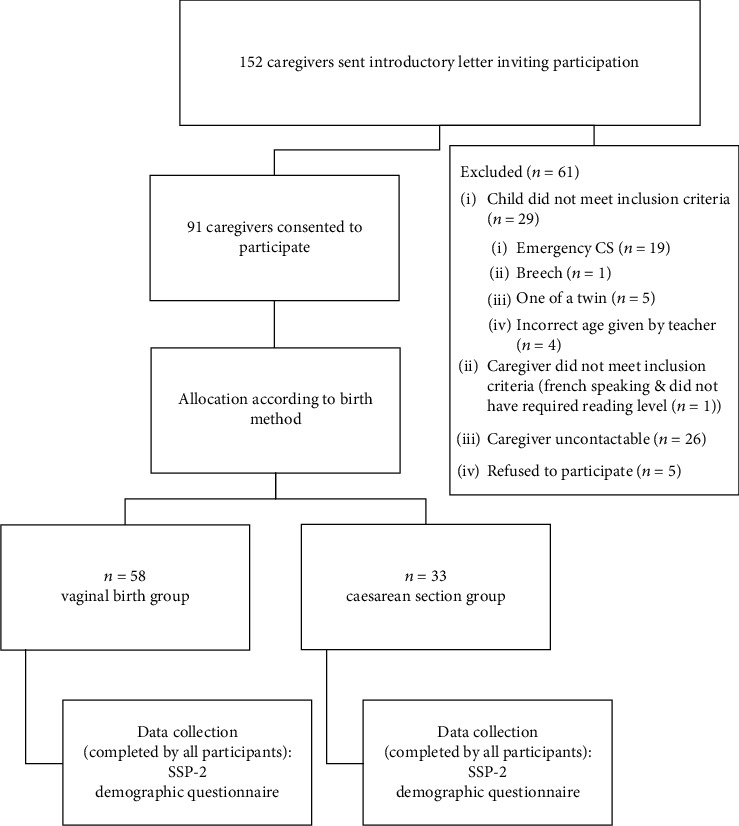
Participant flow chart.

Ninety-one child-caregiver dyads (see Figure 1 for details of the participant flow) completed the SSP-2 and demographic questionnaire. There was no missing data for the SSP-2, but there was some missing demographic data, either because the caregiver did not know the information or could not remember it.

### 3.2. Participant Profile

The characteristics of the mothers according to birth method group are presented in Table 1.

Three maternal demographic variables showed a statistically significant difference when comparing the two birth method groups. Mothers in the VB group were significantly younger (median = 26.0 years) than the CS group (median = 32.0 years; *p* = <0.001). More mothers in the CS group were married (64%; *p* = 0.037), with 57% of the VB group mothers either single or cohabiting. Regarding income level (*p* = 0.003), a higher percentage of mothers in the VB group was from the middle- and low-income groups.

The demographic characteristics of the children according to birth method group are presented in Table 2.

Three child variables demonstrated statistically significant differences between the birth method groups. Gestational age (GA) was higher in the VB group (median = 40 weeks), compared to the CS group (median = 39 weeks; *p* = 0.001). Time from birth to the first breastfeed (measured in minutes) showed almost immediate sucking in the VB group (median = 1 minute), compared to the CS group (median = 12 minutes; *p* = 0.006). There was a higher frequency of sleeping difficulties in the CS group (27%) compared to the VB group (21%; *p* = 0.003).

### 3.3. Prevalence of SHR

The prevalence of SHR, and a description of sensory modulation patterns (including sensory avoiding, sensory seeking and low registration) across the entire sample, and by birth method, is presented in Table 3 [27]. SHR is representative of children who scored +1 (more than) and +2 SD (much more than) others.

The prevalence of SHR in the total sample was 22%. The prevalence of SHR was significantly higher in the VB group (29%) compared to the CS group (9%; *p* = 0.034). The other three categories of sensory modulation disorder all showed a statistically significant higher prevalence in the VB group (sensory avoiding 36%, *p* = 0.006; sensory seeking 48%, *p* = 0.002; and low registration 24%, *p* = 0.043).

### 3.4. Socioeconomic Influences

To understand the unexpected results between the socioeconomic status of the participants, birth method, and SHR, possible associations were explored between these three variables.  Table 4 reports the results examining the association between income level and birth method. This indicated that 74% of mothers in the middle- and low-income level groups had a VB compared with 36% in the high-income level group.

Next, the prevalence of SHR according to income level was examined and is reported in Table 5. In the high-income level group, the total percentage of SHR was 16%. The total SHR for the middle- and low-income groups combined was 24%.

## 4. Discussion

### 4.1. Participant Profiles and Variables Linked to SHR

Mothers in the VB group were significantly younger at the time of giving birth, were majority low- to middle-income status, and were single or unmarried. These demographic characteristics align with prior studies reporting higher rates of CS among older mothers [6], associations between younger maternal age and lower income levels [31], and associations between VB method and lower socioeconomic status in the South African population [32]. The slightly shorter GA in the CS group was an expected finding, as most CSs are planned to take place at 37 to 38 weeks of gestation [33]. The statistically significant difference in minutes to the first breastfeed favouring the VB group correlated with Nissen et al. [34], who similarly identified that the first breastfeed was significantly later in CS deliveries. No overt factors could be linked to the significantly higher frequency of difficulties with sleep in the CS group. However, the first author hypothesises that sleep difficulties could be associated with lower GA and the delays in the first breastfeed in the CS group [27]. Babies who are born at a lower GA struggle to regulate physiological states such as sleeping, and this leads to greater dysregulation and irritability [35]. They are also known to have more frequent respiratory and gastrointestinal problems [33], which may also affect sleep.

### 4.2. Prevalence of SHR

The prevalence of SHR has been studied in a limited number of countries and shows some differences. Prevalence in the United States of America has been reported as 17% in one study [22] and 21% in another [36]. In a study conducted in Saudi Arabia, the prevalence was reported as 31% [37]. In the only South African study measuring prevalence of SHR, the participants were all of low socioeconomic status residing in rural areas of the Western Cape province. A prevalence of 35% was reported [38]. In the first author's study, the prevalence of SHR in the total study sample was 22%, including both birth method types and participants of all socioeconomic groups.

The hypothesis that there would be a higher prevalence of SHR in children born by elective CS compared to those born by VB was rejected, with the VB group having a significantly greater prevalence. Links between socioeconomic status, birth method, and SHR were explored post hoc. These revealed that a high percentage (74%) of mothers in the middle- and low-income group had a VB, and conversely, a high percentage of mothers in the high-income group had a CS. Socioeconomic factors may influence many aspects of maternal and child health care. In the South African context, this was particularly so, since a large proportion of the population was of low socioeconomic status and that there was no universal health care. The socioeconomic status of the study participants largely determined whether mothers accessed private or public health care. Private health care was characterised by better maternal health care, antenatal care, better medical facilities, and higher quality of care than that generally available in public health care. The private health care facilities were therefore likely to result in better health and developmental outcomes. The public health guidelines preclude a CS without a medical reason [9]. A mother participating in the study who was of middle-low socioeconomic status was more likely to have used public health facilities and therefore more likely to have a VB.

The SHR prevalence was higher in the middle-low-income group (24%) when compared to the high-income group, where it was 16%. This higher prevalence in the middle-low-income group correlated with international studies reporting higher rates of SHR in low socioeconomic communities, which found low socioeconomic indicators to be risk factors for developing SHR [20]. Poverty is frequently characterised by poor nutrition, poor sanitation and hygiene, poor maternal education, poor maternal health, chaotic home routines, and lack of stimulation. These factors could all interact to negatively impact on sensory modulation and increase the likelihood of SHR [22, 39].

In the South African context from which these study participants were drawn, a mother from a middle-low-income group would therefore be likely to use public health care facilities and would be more likely to have a VB, and due to the characteristics of a low socioeconomic environment described above, they would be more likely to have a child with SHR.

### 4.3. Additional Factors Influencing SHR in an Upper-Middle-Income Country

Three additional factors noted in the low socioeconomic environments may have contributed to the increase in SHR in the VB group, 74% of whom were of low socioeconomic status. These were the validity of the questionnaire used to assess SHR, the quality and quantity of environmental sensory input, and the level of routine and organization in the child's life.

Firstly, there were challenges to the validity of the questionnaire which were addressed prior to and at the start of data collection. However, even with these two rounds of evaluation and adaptation, challenges were still experienced by some middle- and low-income participants. Most middle- and low-income caregivers took longer to complete the questionnaire. This was attributed to the questionnaire not being in their home language, caregivers' lower educational levels, with resultant poor reading comprehension skills, challenges relating to contextual relevance of some of the statements, and difficulties understanding the Likert scale. This resulted in the questionnaire taking approximately half an hour for these caregivers to complete as opposed to the 15 minutes estimated in the SP-2 manual [5]. It is therefore possible that the validity of the questionnaire was adversely affected for these participants.

Secondly, several environmental factors noted during data collection heightened both the quantity and quality of sensory stimulation experienced by the child and caregiver participants. The first author observed heightened sensory stimulation, especially in the tactile, olfactory, and auditory senses during data collection in the middle- and low-income level environments. The intensity of the stimuli in these environments was linked to overcrowding in homes (which were often inhabited by many members of the extended family), poor sanitation, and inadequate provision of public utilities such as water, electricity, and water-borne sewerage [18]. Cramped conditions in living spaces, often linked with busy schedules, and the absence of routine in daily activities further exacerbated the impact of the intense stimuli described above. No studies could be found that addressed sensory overstimulation in low socioeconomic environments. However, some studies have explored sensory overstimulation of babies in neonatal intensive care units and the increased prevalence of sensory modulation difficulties in these children [40, 41]. Many of the babies in these studies would have been born preterm with less mature nervous systems compared to many of the children taking part in this study. Statements about potential similarities in these populations should therefore be made with caution. It is nevertheless possible that factors of sensory overstimulation may have a similar impact on sensory modulation for children in low socioeconomic environments. This needs to be explored further.

Thirdly, chaotic schedules, lack of routine, inconsistent sleeping and mealtime routines, and resultant high levels of unpredictability have been described by Ursache and Noble [42, 43] as common characteristics of low socioeconomic environments. This sense of disorganization can negatively impact the development of sensory modulation by increasing stress and cortisol levels in the child, decreasing the ability to self-regulate.

### 4.4. Strengths and Limitations

This study contributes to a small number of South African studies exploring SHR, providing valuable additional understanding of its prevalence in a sample representing diverse socioeconomic groups in an upper-middle-income country. To our knowledge, this is also the first study to explore birthing methods as a possible factor in the aetiology of SHR.

The sample size was relatively small, limiting generalisability, and the two birth method groups were not equally weighted, making comparisons less accurate. Language, cultural factors, low education levels, and difficulty reading with understanding impacted on participants' ability to respond to the Likert rated questions asked in the SSP-2. Lack of cultural and contextual appropriateness of the SSP-2 was evident with some statements not being applicable to the participating caregivers. This resulted in the administration of the caregiver questionnaire not being standardised across the various language, cultural, educational, and reading levels. These factors are likely to have impacted the validity of the results, making it difficult to test the hypothesis accurately. The effect of poverty on neurocognitive and behavioural development, as well as associated environmental factors (high levels and intensity of sensory inputs and inconsistent routines), could also be contributing to the higher prevalence of SHR in the middle- and low-income level groups in this study. While the results would have been strengthened if additional clinical observations or physiological measures had been used [22], neither of these was feasible within the context of this study.

The results reported of a higher prevalence of SHR in the VB group may be linked to the many challenges experienced in this study related to contextual factors rather than due to the VB. These challenges can inform future research, for which the following suggestions are made:

1. Research to explore the adaptation of an existing assessment or the development of a new assessment for use in the South African context which would allow for the accurate identification of SHR
2. Repetition of this study using a contextually relevant and culturally appropriate assessment
3. Research which more specifically explores the association between socioeconomic status and SHR

## 5. Conclusion

It was hypothesised that there would be a stronger presence of SHR in children born by CS. However, this was not the case, with a higher prevalence of SHR in the VB group. In the absence of other studies, this study provides the first understanding of patterns of SHR in relation to birth method in a diverse population. The substantial challenges experienced by the caregivers of children from low socioeconomic backgrounds in completing a standardised assessment tool likely affected the accuracy of their responses on the SSP-2 and thus the correct identification of SHR difficulties. In addition, poverty and environmental factors may have contributed to the higher SHR in the VB group of children. The presentation in this paper of the significant challenges collecting data in a diverse population could be helpful for researchers in similar contexts and guide further areas of research into other factors that may be linked to higher SHR in diverse populations.

## Figures and Tables

**Table 1:** Demographic characteristics of the mothers per birth method group (*N* = 91).

Variable	Vaginal birth group (*n* = 58)		Caesarean section group (*n* = 33)				
	Median (range)	Missing data No. (%)	Median (range)	Missing data No. (%)	Mann–Whitney *U*		*p* value
Maternal age	26.0 (17.0-43.0)	0 (0)	32.0 (17.0-44.0)	2 (6)	499.0		<0.001^∗^
	No. (%)		No. (%)		Chi-square	df	*p* value
Income level					11.49	2	0.003^∗^
Low	24 (41)	0 (0)	8 (24)	0 (0)			
Middle	25 (43)		9 (27)				
High	9 (16)		16 (48)				
Marital status							0.037^∗^^#^
Widow	0 (0)	0 (0)	2 (6)	0 (0)			
Single	21 (36)		7 (21)				
Divorced	1 (2)		0 (0)				
Co-habiting	12 (21)		3 (9)				
Married	24 (41)		21 (64)				
Education level		0 (0)		2 (6)	7.68	7	0.362
Grade 0-11	25 (43)		9 (27)				
Grade 12 and technical	23 (40)		10 (30)				
Tertiary	10 (17)		12 (36)				
Stress^1^		0 (0)		0 (0)			1.000^#^
Yes	9 (16)		5 (15)				
No	49 (84)		28 (85)				
Illness		0 (0)		0 (0)			
Yes	6 (10)		4 (12)				1.000^#^
No	52 (90)		29 (88)				
Smoking		0(0)		0(0)	2.52	1	0.113
Yes	8 (14)		9 (27)				
No	50 (86)		24 (73)				
Alcohol		0(0)		0(0)			0.487^#^
Yes	5 (9)		5 (15)				
No	53 (91)		28 (85)				
Recreational drugs		0 (0)		0 (0)			1.000^#^
Yes	1 (2)		1 (13)				
No	57 (98)		32 (97)				
High blood pressure^2^						1	0.795
Yes	12 (21)		6 (18)	1 (3)	0.067		
No	45 (78)	1 (2)	26 (79)				

^∗^Statistically significant difference *p* < 0.05. ^#^Fisher's exact test, two-tailed. ^1^Stress measured through a single question to obtain the mother's subjective opinion. ^2^High blood pressure: Yes = mother used medication during pregnancy for hypertension; No = mother did not use medication during pregnancy for hypertension.

**Table 2:** Demographic characteristics of the children per birth method group (*N* = 91).

Variable	VB group (*n* = 58)		CS group (*n* = 33)		Mann–Whitney *U*		*p* value
	Median (range)	Missing data	Median (range)	Missing data			
Birth weight	3.4 (1.8-4.7)	11 (19)	3.2 (1.3-4.5)	4 (12)	596.0		0.366
Gestational age	40 (29-42)	3 (5)	39 (32-42)	4 (12)	472.5		0.001^∗^
Minutes to first contact	1 (1-38880)	3 (5)	1 (1-360)	4 (12)	765.5		0.765
Minutes to first breastfeed	1 (1-38880)	6 (10)	12 (1-360)	4 (12)	478.0		0.006^∗^
Age to first solids	6 (1-12)	7 (12)	6 (1-8)	2 (6)	758.5		0.761
Pressure^1^ in months	12 (0-54)	4 (7)	18 (1-43)	6 (18)	645.5		0.405
	No. (%)		No. (%)		Chi-squared	df	*p* value
Gender		0(0)		0(0)	0.29	1	0.590
Female	30 (52)		19 (58)				
Male	28 (48)		14 (42)				
Birth order		1 (2)		1 (3)			0.830^#^
First	23 (40)		9 (27)				
Second	18 (31)		13 (39)				
Third	12 (21)		8 (24)				
Fourth and fifth	4 (6)		2 (6)				
Colic^2^		0 (0)		0 (0)	0.16	1	0.693
Yes	12 (21)		8 (24)				
No	46 (79)		25 (76)				
Illness^3^		0 (0)		0 (0)			0.133^#^
Yes	3 (5)		5 (15)				
No	55 (95)		28 (85)				
Ear infections^3^		0 (0)		1 (3)			0.780^#^
Yes	11 (19)		5 (15)				
No	47 (81)		27 (82)				
Allergies^3^		0 (0)		1 (3)			1.000^#^
Yes	7 (12)		3 (9)				
No	51 (88)		29 (88)				
Eczema^3^		0 (0)		0 (0)	1.01	1	0.314
Yes	16 (28)		6 (18)				
No	42 (72)		27 (82)				
Asthma^3^		0 (0)		0 (0)			0.718^#^
Yes	5 (9)		4 (12)				
No	53 (91)		29 (88)				
Injuries^3^		0 (0)		0 (0)			0.129^#^
Yes	0 (0)		2 (6)				
No	58 (100)		31 (94)				
Eating problems^2^		0 (0)		0 (0)			0.349^#^
Yes	2 (3)		3 (9)				
No	56 (97)		30 (91)				
Crying^2^		0 (0)		0 (0)	0.51	1	0.474
Yes	12 (21)		9 (27)				
No	46 (79)		24 (73)				
Sleeping problems^2^		0 (0)		0 (0)			0.003^∗^^#^
Yes	12 (21)		9 (27)				
No	46 (79)		24 (73)				

^∗^Statistically significant difference *p* < 0.05. ^#^Fisher's exact test, two-tailed. ^1^Pressure was defined as swaddling, being carried in a traditional manner on the caregiver's back, or in a baby carrier or sling, and was estimated by the mother in months of the baby's life that these methods were used. ^2^Colic, eating problems, crying and sleeping problems: measured through a question to obtain the mother's subjective opinion. ^3^Illnesses, ear infections, allergies, eczema, asthma, and injuries: based on a medical diagnosis; Yes = this was in the child's history as reported by caregiver; No = this was not in the child's history as reported by caregiver.

**Table 3:** Patterns of sensory modulation disorders (*N* = 91).

SMD categories	Vaginal birth group (*n* = 58)	Caesarean section group (*n* = 33)	Total group (*N* = 91)	*p* value (Fisher's exact test)
	No. (%)	No. (%)	No. (%)	
Sensory hyperreactivity				
Yes^1^	17 (29%)	3 (9%)	20 (22%)	0.034^∗^
No^2^	41 (71%)	30 (91%)	71 (78%)	
Sensory avoiding				
Yes^1^	21 (36%)	3 (9%)	24 (26%)	0.006^∗^
No^2^	37 (64%)	30 (91%)	67 (74%)	
Sensory seeking				
Yes^1^	28 (48%)	15 (45%)	43 (47%)	0.002^∗^
No^2^	30 (52%)	18 (55%)	48 (53%)	
Low registration				
Yes^1^	14 (24%)	2 (6%)	16 (17%)	0.043^∗^
No^2^	44 (76%)	31 (94%)	75 (83%)	

^∗^Statistically significant difference *p* < 0.05. ^1^Yes = the child scored 1 (more than) or 2 (much more than) standard deviations above the mean. ^2^No = the child scored 0 SD (typical performance).

**Table 4:** Method of birth by income level (*N* = 91).

Birth method	Low- and middle-income levels (*n* = 66)	High-income level (*n* = 25)
	No. (%)	No. (%)
Vaginal birth group	49 (74%)	9 (36%)
Caesarean section group	17 (26%)	16 (64%)

**Table 5:** SHR SD per income level group.

Income level	SD 0^1^	SHR SD +1^2^	SHR SD +2^3^
	No. (%)	No. (%)	No. (%)
High (*n* = 25)	21 (84)	4 (16)	0 (0)
Middle and low (*n* = 66)	50 (76)	13 (20)^∗^	3 (5)^∗^

^1^SD 0 = typical performance. ^2^SD +1 = “more than others”, having more symptoms of dysfunction than typically performing children. ^3^SD +2 = “much more than others”, with the child having “much more” in terms of symptoms of dysfunction than typically performing children. ^∗^ When SHR +1 and +2 were calculated individually, the percentages were 20% and 5%, respectively. When calculating the sum of SHR +1 and +2, the statistician advised to total the raw data (13 + 3 = 16) and then calculate the percentage from this total, which is 24%. This discrepancy between the table figures and the text occurred due to the rounding of the percentages.

## Data Availability

The data is available from the first author on request.
